# Changing Epidemiology of Rotavirus-Related Hospitalizations in Rio De Janeiro, Brazil, from 2002 to 2006

**DOI:** 10.2174/1874357900701010047

**Published:** 2007-12-31

**Authors:** Vera S Gouvea, André L.S Domingues, Felipe G Naveca, Adriana R Pedro, César C Bevilacqua

**Affiliations:** 1 Instituto de Microbiologia, Universidade Federal do Rio de Janeiro, Rio de Janeiro, Brazil; 2 Instituto de Puericultura e Pediatria Martagão Gesteira, Universidade Federal do Rio de Janeiro, Rio de Janeiro, Brazil

**Keywords:** Rotavirus diarrhea, hospitalizations, genotypes/electropherotypes, vaccine.

## Abstract

A prospective hospital-based sentinel study conducted in Rio de Janeiro identified a shift in the pattern (long to short electropherotype and P(8) to P(4) genotype) of rotavirus strains recovered from children with severe diarrhea a few months after the far-reaching Brazilian rotavirus immunization program was launched, posing new interesting challenges.

## INTRODUCTION

Group A rotavirus infection is an important cause of pediatric hospitalization due to severe diarrhea and a global public health concern. Rotaviruses possess a genome of 11 segments of double-stranded RNA that may undergo natural reassortment during mixed infections [1]. According to the composition of the genomic ensemble, rotavirus strains are classified into genogroups [2]. One major (Wa-like) and two minor (DS1- and AU1-like) genogroups have been described for human strains, with those of Wa and AU1 genogroups usually associated with long electropherotypes and those of DS1 genogroup with short ones. Rotavirus genomic segments 4 and 9 code for the outer capsid VP4 (P) and VP7 (G) proteins, respectively, which independently determine the virus serotypes [3]. Although immunity against rotavirus infection is still poorly understood, protection against severe diarrheal disease is believed to be serotype-specific, and vaccines targeting some of the most common human rotavirus serotypes, G1, G2, G3, G4 and P(8), have been developed by two different approaches [4]. One was based on the production of several animal-human monoreassortants containing the desired human gene 9 or 4 segment and the animal genomic backbone; the other was based on the classical attenuation of a rotavirus strain. In August 1998, the tetravalent rhesus-human reassortant rotavirus vaccine, RRV-TV, Rotashield^®^ (Wyeth Laboratories, USA) was licensed in the United States and included in the immunization schedule for infants. After one year however, rotavirus immunizations were suspended and the vaccine was withdrawn from the market due to its association with intussusception in vaccinated recipients [5, 6]. Seven years later, in August 2005, an oral live attenuated human G1P(8) rotavirus vaccine, Rotarix^®^ (GlaxoSmithKline, Belgium) was licensed for private pediatric use in Brazil and by March 2006, it was incorporated into the National Childhood Immunization Program assuring free vaccination to all 2-month old infants in the country [7, 8].

Rotavirus vaccines aim at preventing infant deaths and hospitalizations due to severe diarrhea. Thus, prospective hospital-based studies on rotavirus-associated hospitalizations are critical to assess changes related to immunization programs. Anticipating vaccine introduction, a sentinel study was established at the Emergency Service of our university children’s hospital to investigate the burden of acute childhood gastroenteritis and to gather information on rotavirus strains before and after vaccine introduction. Here we report the results on the rotavirus strains causing the most severe diarrheal cases that required at least an overnight hospital stay for intravenous rehydration therapy.

## MATERIALS AND METHODS

The Instituto de Puericultura e Pediatria Martagão Gesteira (IPPMG/UFRJ) serves a low socioeconomic population living in nearby crowded communities adjacent to the university campus in Rio de Janeiro. The study was approved by the hospital Ethical Committee (Proc#08/2002) and fecal specimens, clinical and epidemiological data were collected after written informed consent from the child’s parent or guardian. Total RNA was purified from fecal specimens by adsorption and elution from hydroxyapatite [9] and screened for rotavirus genomic segments by polyacrylamide gel electrophoresis (PAGE) [10] and by reverse transcription-PCR (RT-PCR) followed by nested PCR with pools of G and P type-specific primers [11, 12]. Typing results were confirmed in a dot hybridization assay [13].

## RESULTS

**Fig. (1):**
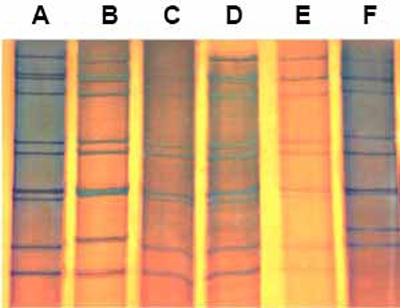
PAGE of group A rotavirus 10 dsRNA genome with the typical tight triplet formed by segments 7, 8 and 9. Electropherotypes: L1 (A), L4 (B), L9 (C), L10 (D), L8 (E), and S1 (F).

**Fig. (2):**
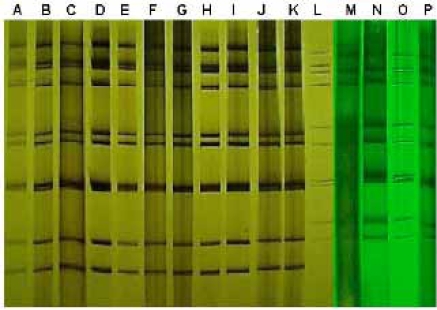
PAGE of rotaviruses showing the same long electropherotype L6 found in specimens from 2003 (A), 2004 (B-C), 2005 (D-J) and 2006 (K), and the distinct short patterns S3 (L), S2 (M-N), S4 (O) and S1 (P) obtained in 2006.

From August 2002 to December 2006, rotavirus was detected in 97 (50%) out of 193 specimens (Table **1**). One strain showed an atypical profile and was confirmed as a group C rotavirus by RT-PCR [9]. An electropherotype was discernible in 88 (92%) specimens and eight were identified only by the more sensitive genotyping assay (Figs. **1**,**2**). Mixed infections were revealed in 23 (24%) specimens by genotyping, and in seven (7%) by PAGE including two specimens in which only a single GP combination was detected thus, raising to 25 (26%) the total number of mixed infections observed. Major electropherotypes, such as L1 or L6, were recognizable in some mixtures despite the presence of additional bands, but other specimens that contained multiple rotavirus strains (multiple G or P types) presented unique electropherotypes without extra bands [14]. The vast majority (92%) of the specimens contained G1 or G9 strains, or both, although there was a considerable year to year strain variation. Rotavirus G1P(8) profile L1 was detected in 27% of the specimens, either singly or with other rotavirus strains, during all years except 2005 when an emerging G9P(8) profile L6 strain overran other strains and accounted for practically all confirmed rotavirus-related hospitalizations of that year. Overall, genotype G9 was detected in 66% and P(8) in 72% of the rotavirus infections. Types G1 and G9 were also found in combination with P(6) and P(4), either in single or multiple infections; G4 was found only in mixtures and mostly in 2004; G2 was found sporadically and presented short electropherotypes when combined with P(4); and a rare G3P(9) was detected in 2002. The two major P(8) strains (long profiles L1 and L6) were also seen in the summer of 2006, but only conspicuous short profiles were detected after April 2006. Those emerging strains of short profiles were typed as G2P(4), G9P(4) and G2G10P(4).

Considerable variation was also observed in the temporal distribution of rotavirus strains. Although this one-site surveillance study covered only the last semester of 2002, it showed a great variety of strains and a very high rotavirus detection rate concentrated in August, the first month of the study and mid winter in Rio de Janeiro. The following two years, 2003-2004, showed a marked decrease in strain diversity, a tendency that was confirmed in the very unusual year of 2005 when a single strain predominated (Table **1**, Fig. **2**). In this atypical year, an overall marked drop in rotavirus detection rate was observed among hospitalized children and most of the rotavirus-associated cases occurred earlier, in the spring months of March and May. Temporal displacement of rotavirus activity occurred again in 2006, when strains L1 and L6 were detected in the summer months of January-March. Only P(4) strains of short profiles were detected sporadically thereafter and the expected winter peak of rotavirus activity was not observed.

The majority (88%) of the rotavirus-associated cases comprised infants and children less than two years of age with the 6-12 month-old group being the most affected (Table **2**). These results are in agreement with the highest incidence of dehydrating disease recorded in developing countries [8, 14] and with the recommendation for rotavirus immunization of infants at the early age of 6-14 weeks old [14, 15].

## DISCUSSION

Great diversity and complex mixtures of strains were described as hallmarks of the epidemiology of rotavirus infections in southern Brazil where rotavirus infections occur year round with unpredictable peaks of activity [1, 14, 16, 17]. This study showed those features and identified interesting changes in the epidemiology of rotavirus strains causing the most severe diarrheal cases over a period of four and a half years. In spite of the modest number of specimens available in a single-site study dealing only with hospitalized cases, our study captured the general trend observed in similar studies conducted in three other public hospitals in the city during 1996-1998 and 2004 [17, 18]. Those studies reported a great diversity of strains among hospitalized children with diarrhea before 2002 and an overwhelming predominance of G1P(8) and G9P(8) in 2004, similar to the findings reported here. The tendency of a major single epidemic strain to overpower local strains was demonstrated by the predominance of G9P(8) profile L6 strain among the IPPMG-hospitalized children during 2005, as had been previously observed with another G9 strain (HFF16-like) among the most severe diarrheal cases treated at Hospital Fernando Figueira, Rio de Janeiro, during 1998 [13]. Changing in rotavirus epidemiology toward a predominance of G9 strains is a worldwide phenomenon extensively reported by many authors and incorporation of G9 as a fifth G component in future polyvalent vaccines is under consideration [19]. The sudden shift towards strains displaying short profiles and P(4) specificity observed in May 2006 seems to signal the local emergence of antigenically and genetically unrelated strains, most likely of the DS1 genogroup^i^. Indeed, other recent studies conducted at the Salles Netto Municipal Hospital in Rio de Janeiro^ii^ and in the midlands of Minas Gerais^iii^, also reported detecting short profile G2P(4) strains at increasing frequency among hospital-assisted children with diarrhea during 2006 and beginning of 2007. Similar shift from predominantly long to short rotavirus electropherotypes had been observed previously in Paraguay, a neighbor country where rotavirus vaccine has yet to be introduced^iv^. Taken together, those independent studies seem to confirm the proposed trend observed at our sentinel hospital by mid 2006. Whether those changes represent a new trend on rotavirus strains circulating in the South American cone region or they portend a new global trend dictated by the unpredictable fluctuation of rotavirus strains everywhere, must await reports from other continents [15]. Emergence of strains of the third, AU1 genogroup, is a possibility, given the presence of similar strains (G3P(9), profile L3) in the region.

Although no clear link between the introduction of the G1P(8) vaccine and a shift in rotavirus genotypes could be established, the observed changes coincided with the implementation of the rotavirus immunization program in Brazil and might reflect, at least in part, an immediate response to the extensive, virtually mandatory and year-long vaccination of infants with an attenuated strain of the Wa genogroup. Large clinical trials of the Rotarix^®^ vaccine performed in eleven Latin American countries showed high levels of protection not only against homotypic G1P(8) strains (91% vaccine efficacy) but also against strains sharing the P(8) specificity (and probably several other proteins as well) combined with either G3, G4, or G9 serotype (87% efficacy) [7, 8]. The vaccine conferred only low protection (42% efficacy) against rotavirus G2P(4) strains that do not share G or P epitopes with the vaccine strain [8]. Thus, one would expect the vaccine program to induce an early sharp decrease in the number of rotavirus-associated hospitalizations, leaving only a reduced number of severe cases associated to otherwise minor endemic or emerging strains of the non-Wa genogroup. This was the 2006 scenario observed in the present sentinel study, as the short P(4) rotavirus strains did not produced major outbreaks or an accentuated epidemic season, but rather, appeared to represent highlighted background. However, this situation might change drastically in the following years along with the changing rotavirus ecology and an ever growing population of vaccinated Brazilian children. This unique situation poses a great opportunity to evaluate vaccine performance facing rotavirus infections with strains of distinct genotypes and distinct genogroups, to examine the contribution of rotavirus genes other than the VP4- and VP7-encoding genes to rotavirus immunity or susceptibility to severe diarrheal disease, as well as to assess the duration of vaccine protection. This single-site four and a half-year study included the first nine months of the far-reaching Brazilian rotavirus immunization program, stressed the dynamic nature of rotavirus infections in the city, and further demonstrated the usefulness of electropherotyping to perceive new trends in rotavirus epidemiology.

## Figures and Tables

**Table 1.** G and P Genotypes and Electropherotypes of the 97 Rotavirus Positive Samples Out of 193 Specimens from Children Hospitalized at IPPMG from August 2002 to December 2006.

Genotype	E-Type^a^	No. of Specimens					
		2002	2003	2004	2005	2006	Total
G1P(8)	L1	4	3	6	-	-	13
G1P(6)	nd	-	1	-	-	-	1
G1P(4)	nd	-	1	-	-	-	1
G2P(4)	S1, S3	2	-	-	-	4	6
G3P(9)	L3	1*	-	-	-	-	1
G9P(8)	L6	-	4	11	24	3	42
G9P(6)	L10	-	-	1	1	-	2
G9P(4)	S2	1*	-	-	-	6	7
G1G2P(8)	L1	-	-	3*	-	-	3
G1G4P(8)	L1	-	-	3	-	-	3
G1G9P(8)	L1	1	3	-	-	2	6
G1G9P(4)	L8	-	2	-	-	-	2
G2G10P(4)	S4	-	-	-	-	1	1
G4G9P(8)	L4	1	-	-	-	-	1
G1G4G9P(8)	L6	-	-	1*	-	-	1
G1P(8)P(6)	L2	1*	-	-	-	-	1
G9P(8)P(4)	L5	1*	-	-	-	-	1
G9P(8)P(6)	L6	-	-	-	2	-	2
G1G4P(8)P(6)	L9	-	-	1	-	-	1
G1G9P(8)P(4)	L1	-	1*	-	-	-	1
Total Rotavirus: Group A		12	15	26	27	16	96
Group C	L7	-	1	-	-	-	1
Distinct E-types (N)		(7)	(4)	(4)	(2)	(5)	(14)
Mixed infections (%)		6 (50)	6 (40)	8 (31)	2 (7)	3 (19)	25 (26)
Specimens analyzed (% Rotavirus positive rate)		14 (86)	25 (64)	46 (56)	79 (34)	29 (55)	193 (50)

a Electropherotypes L: long, S: short, and nd: not defined.

* One sample in the group presented the designated electropherotype plus one or more extra bands.

**Table 2.** Age Distribution of the Group A Rotavirus-Associated Diarrheal Cases Hospitalized from August 2002 to December 2006

Age (Months)	No. of Cases Per Year						
	2002^a^	2003	2004	2005	2006	Total	(%)
< 6	1	2	3	7	5	18	(19)
6 - 12	6	10	15	12	6	49	(51)
13 - 24	2	2	6	5	2	17	(18)
25 - 60	2	1	2	3	1	9	(9)
61 - 120	1	0	0	0	2	3	(3)

a Specimen collection started in August.

## Footnotes

i Pedro AR, Dias GS, Gomes SP, Chinem ESS, Gouvea V. Acute gastroenteritis in a pediatric hospital in Rio de Janeiro: shift in rotavirus strain prevalence during 2006. Virus Rev Res 2007; 12(Suppl): 164.

ii Carvalho-Costa FA, Araújo IT, Assis RMS, et al. Rotavirus surveillance in Rio de Janeiro, Brazil, during 2005-2007: reemergence of G2P(4) genotype. Virus Rev Res 2007; 12(Suppl): 164.

iii Cruz RL, Morais ATS, Gouvêa VS, Domingues ALS. Rotavirus-associated infantile diarrhea in Uberaba, Minas Gerais. Virus Rev Res 2007; 12(Suppl): 165.

iv Amarilla AA, Espínola EE, Galeano MF, Farina N, Russomando G, Parra GI. Epidemiological pattern of rotavirus infections in Paraguayan population from 2004 to 2005: detection of rotavirus strains with short RNA pattern at high incidence in children. Virus Rev Res 2006; 11(Suppl): 123.
